# A simple method using CRISPR-Cas9 to knock-out genes in murine cancerous cell lines

**DOI:** 10.1038/s41598-020-79303-0

**Published:** 2020-12-18

**Authors:** Airi Ishibashi, Kotaro Saga, Yuuta Hisatomi, Yue Li, Yasufumi Kaneda, Keisuke Nimura

**Affiliations:** Division of Gene Therapy Science, Department of Genome Biology, Graduate School of Medicine, Osaka University, 2-2 Yamada-oka, Suita, Osaka 565-0871 Japan

**Keywords:** Biotechnology, Cell biology, Genetics

## Abstract

CRISPR-Cas9 system can be used to generate knock-out cancer cell lines. An insertion or deletion induced by a single guide RNA (gRNA) is often used to generate knock-out cells, however, some cells express the target gene by skipping the disrupted exon, or by using a splicing variant, thus losing the target exon. To overcome this unexpected expression of the target gene, almost the entire gene can be swapped with a selection marker. However, it is time-consuming to create a targeting vector which contains 5′ and 3′ homology arms flanked by a selection marker. Here, we developed a simple and easy method called SUCCESS (Single-strand oligodeoxynucleotides, Universal Cassette, and CRISPR/Cas9 produce Easy Simple knock-out System), to knock-out a target gene without constructing a targeting vector. Our method removed the targeted large genomic region by using two pX330 plasmids encoding Cas9 and gRNA, two 80mer single strand oligodeoxynucleotides (ssODN), and a blunt-ended universal selection maker sequence in B16F10 murine cancer cell and ID8 murine ovarian cancer cell. SUCCESS generated knock-out clones in two murine cancer cell lines by homozygous deletion of the target genomic region, and without constructing targeting vectors. Thus, our method can be widely applied to generate homozygous knock-out cell lines, as well as knock-in cell lines.

## Introduction

Gene knock-out (KO) is a critical method for identifying the functions of coding and non-coding genomic regions. Recent emergence of genome editing tools such as CRISPR/Cas9 can be used to homozygously introduce double-strand breaks in target genomic regions^1–4^. Non-homologous end-joining (NHEJ) fixes these double-strand breaks, but results in insertion-deletions (INDELs) during the process. INDELs in target exons generate a premature termination codon (PTC) in mRNA by changes in the reading-frame, which induces degradation of nascent mRNAs with a PTC by the nonsense-mediated decay (NMD) system^5^. Thus, the introduction of INDELs in exons leads to disrupted protein expression. However, several genes with a single INDEL can continue to express proteins by exon skipping to remove the burden exon, or by using a different in-frame ATG after the INDEL^6,7^.

To avoid unexpected protein expression from the target gene, deleting almost the entire gene is advantageous when creating KO cells. Conventional strategy to make creating KO cells requires a targeting vector with a selection marker flanking the 5′ and 3′ homology arms; however, constructing targeting vectors is time-consuming. Another strategy was developed to insert exogenous DNA sequences into genomes using short single-strand oligodeoxynucleotides (ssODNs)^8^. While ssODNs act as “glue” to ligate DNA ends, it is still unclear if ssODNs can efficiently insert exogenous DNA into genomes to establish KO cells in cancerous cell lines harboring aberrantly increased number of chromosomes.

In this study, we used a simple and easy method called SUCCESS (Single-strand oligodeoxynucleotides, Universal Cassette, and CRISPR/Cas9 produce Easy Simple knock-out System) to delete a genomic region by using two plasmids coding Cas9 and gRNA, two 80mer-ssODNs, and a blunt-end DNA coding selection marker in B16F10 murine melanoma cells with 77 chromosomes^9^. A high antibiotic dose was crucial for efficient homozygotic knock-in (KI) of the selection marker. ssODN facilitated the expected ligation at the 3′ terminus of the selection marker but not at the 5′ terminus. The shape of the ends of the DNA coding selection marker was critical for promoting efficient homozygotic KI of the selection marker. Multiple selection markers did not enhance the efficiency of the homozygotic KI; instead, they decreased the efficiency of the expected DNA ligation between the genome and selection marker. SUCCESS also generated a KO clone in ID8 murine ovarian cancer cells. Our results confirmed that SUCCESS is a simple method to establish homozygously deleted full length genes from cell lines without a need for constructing a targeting vector.

## Results

### Overall strategy

**Figure 1:**
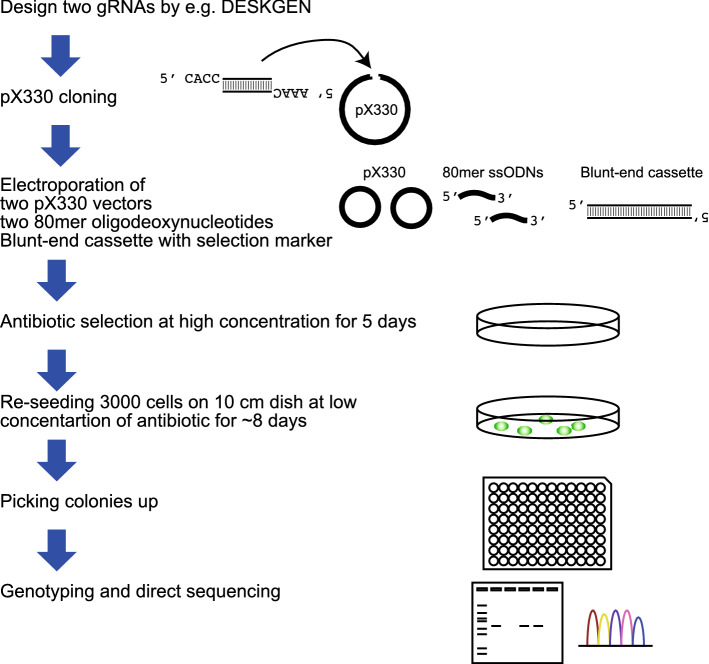
The schema of SUCCESS. SUCCESS requires the construction of only 2 pX330 plasmids and 2 single-strand oligodeoxynucleotides (ssODNs) to delete the full length of the target gene from the genome. The blunt-end cassette with selection marker can be used for any gene knockout.

SUCCESS is based on the CRISPR/Cas9 system to introduce double-strand breaks in the target genomic regions. Two guide RNA (gRNA)s were designed for deleting the full gene length. The target genomic region was deleted by two pX330 plasmids harboring gRNA sequence, two 80mer ssODNs, and a blunt-end cassette with an antibiotic resistance sequence. Single cell clones were formed by re-seeding 3000 cells in a 10 cm dish after high antibiotic selection, then verified by PCR and direct sequencing. The procedure is illustrated in Fig. 1.

### SUCCESS induced homozygotic deletion of the target gene

**Figure 2:**
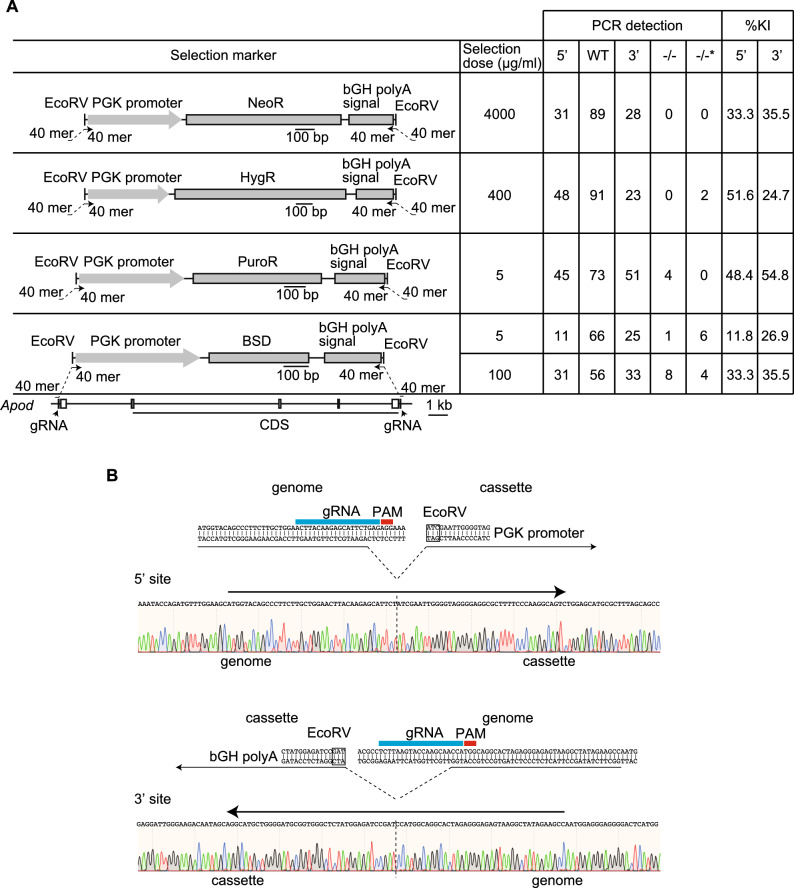
SUCCESS induced homozygotic deletion of the target gene. (**A**) The design of the selection markers is shown. Knock-in efficiency of the selection marker were calculated using genotype results of 93 clones. Arrows show 80mer ssODNs consisting of a 40mer homology sequence to the target genomic region and another 40mer homology sequence to the cassette with selection marker. The tables indicate the results of genotyping of 93 clones. BSD, blasticidin S resistant gene; HygR, hygromycin resistant gene; NeoR, neomycin resistant gene; PuroR, puromycin resistant gene. (**B**) The schema of ligation between the target genomic region and the cassette with selection marker, both at 5′ and 3′ sites. The ligation was analyzed by direct sequencing. 5′, 5′ site between the genomic region and the cassette; 3′, 3′ site between the genomic region and the cassette; WT, wild-type allele; −/−, homozygously knockout clones: −/−*, homozygously knockout clones without the detection of 5′ or 3′ sites ligation between the genomic region and the cassette; %KI, percentage of knock-in in 93 clones.

In establishing SUCCESS to homozygously delete the full length of the target gene from the genome, we first compared antibiotics for selection of clones after introducing two pX330 plasmids encoding Cas9 and gRNA, two ssODNs, and a blunt-end selection marker to delete *Apolipoprotein d* (*Apod*), since ~ 18 kbp of *Apod* gene length was considered appropriate to demonstrate the method and *Apod* is a non-lethal gene^10^ (Fig. 2A). B16F10 cells have 4 copies of chromosome 16, resulting in 4 copies of *Apod*^9^. We first determined the antibiotic doses to induce cell death in wild-type B16F10 cells; almost all B16F10 cells were killed with 2.5 mg/mL neomycin, 400 µg/mL hygromycin, 0.75 µg/mL puromycin, or 4 µg/mL blasticidin S (Fig. S1). Zeocin could not kill wild-type B16F10 cells without inducing morphological changes. High antibiotic dose that did not induce morphological changes was used for five days to select clones that had all *Apod* alleles replaced by the selection markers, to avoid unexpected effects. Insertion of the selection marker into the *Apod* gene was detected in all antibiotic-treated cells (Fig. 2A). *Apod* KO clones were not found in 4000 µg/mL neomycin-treatment cells which showed 33.3% efficiency for 5′ ligation site and 35.5% efficiency for 3′ to knock the selection marker into the target genomic region (Fig. S2A). The KI efficiency of the selection marker in 400 µg/mL hygromycin-treatment was better than that of neomycin-treatment (51.6% for 5′ and 24.7% for 3′ ligation site) (Fig. S2B). Two clones without wild-type alleles were obtained following hygromycin-treatment, but these clones did not have the expected ligation at 5′ and 3′ sites, which is shown as −/−* in Fig. 2A. Five µg/mL puromycin-treatment showed the highest selection marker KI efficiency (48.4% for 5′ and 54.8% for 3′ ligation site), resulting in 4 KO clones with the expected ligation at both sites (Fig. S2C). High blasticidin S concentration at 100 µg/mL resulted in 8 KO clones and 4 KO clones without the expected ligation at both sites (Fig. S2D). Next, we examined if high antibiotic concentration was necessary to increase the efficiency of knocking a selection marker into the target genomic region. Low blasticidin S concentration at 5 µg/mL showed low selection marker KI efficiency (11.8% for 5′ and 26.9% for 3′), resulting in 1 KO clone and 6 KO clones without the expected ligation at both sites, although 5 µg/mL blasticidin S completely killed wild-type cells (Fig. S1). Next, we confirmed the ligation between the end of the genome and the cassette containing the selection marker by direct sequencing of PCR amplicons at 5′ and at 3′ regions in KO clones generated by high blasticidin S-treatment (Fig. 2B). Non-homologous end joining (NHEJ) inhibitor did not change survived cell number after blasticidin S selection (Fig. S2E). Our results showed that selection using 100 µg/mL blasticidin S was highly efficient to delete the target genomic region homozygously by introducing pX330 plasmids, ssODNs, and blunt-end selection marker, and that our method ligated the genome and selection marker as the design of ssODNs.

### The blunt-end of the cassette and ssODNs were crucial for increasing KI efficiency

**Figure 3:**
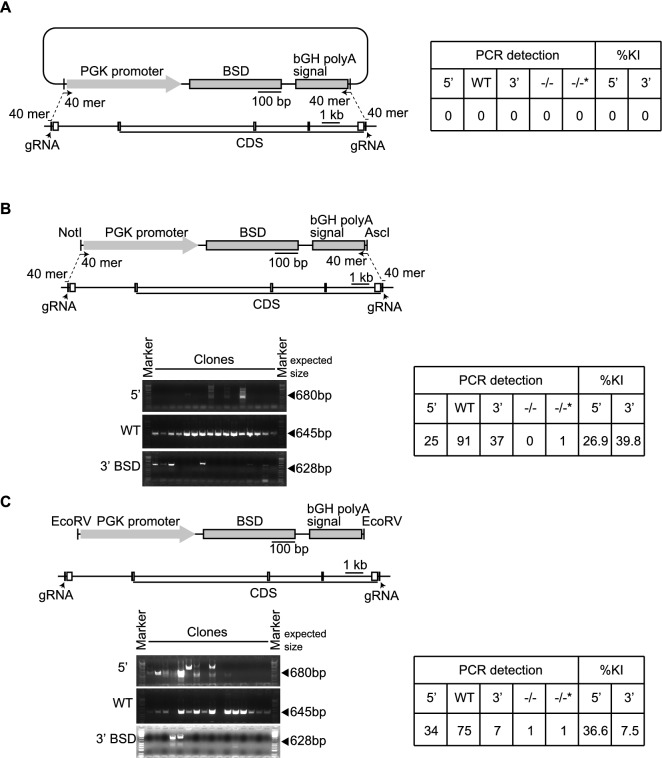
The blunt-end of the cassette with selection marker and single-strand oligodeoxynucleotides (ssODNs) were critical for increasing the efficiency to knock the cassette into genome. (**A**, **B**) The schemas of knockout of the target gene by pX330 plasmids, ssODNs, and un-cut plasmid with selection marker (**A**) or sticky ends of DNA coding selection marker cut by NotI and AscI (**B**). (**C**) The schema of knockout of the target gene by pX330 plasmids and the DNA with selection marker smoothly cut by EcoRV without ssODNs. Agarose gel images showing the genotyping results. The tables indicate the genotyping results of 93 clones. 5′, 5′ site between the genomic region and the cassette; 3′, 3′ site between the genomic region and the cassette; WT, wild-type allele; −/−, homozygously knockout clones: −/−*, homozygously knockout clones without the detection of 5′ or 3′ sites ligation between the genomic region and the cassette; %KI, percentage of knock-in in 93 clones.

Next, we examined if the shape of the end of the DNA coding the selection marker was critical for generating KO clones using high blasticidin S concentration. Although introduction of an un-cut plasmid with a selection marker is known to establish transgenic clones by random integration of the plasmid into the genome^11^, the un-cut plasmid with a selection marker did not allow cells to form colonies under the high antibiotic dose (Fig. 3A). The sticky ends of the DNA coding the selection marker cut by NotI and AscI showed decreased efficiency to knock the cassette into the target genome (26.9% for 5′ and 39.8% for 3′ sites, Figs. 3B and S3A). Further, the insertion efficiency of the cassette containing the selection marker decreased without ssODNs (36.6% for 5′ and 7.5% for 3′ sites, Figs. 3C and S3B). Moreover, the loss of ssODNs or the sticky ends of the DNA coding the selection marker caused disordered ligation between the target genomic region and cassette (Figs. 3B,C, S3A,B). Thus, ssODNs and the shape of the ends of the DNA coding selection marker were critical for promoting the correctly aligned ligation between the target genomic regions and the cassette.

### The homology arms increased the efficiency to knock the cassette with the selection marker into the target genomic region

**Figure 4:**
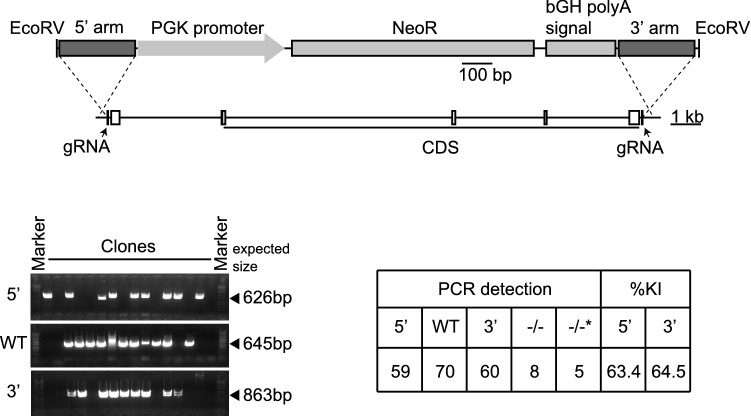
The homology arms improved the efficiency to knock the cassette with selection marker into the target genomic region. The schema of knockout of the target gene by pX330 plasmids and the 250 bp homology arms-flanked selection marker. Agarose gel images show the genotyping results. The table indicates the genotyping results of 93 clones. 5′, 5′ site between the genomic region and the cassette; 3′, 3′ site between the genomic region and the cassette; WT, wild-type allele; −/−, homozygously knockout clones: −/−*, homozygously knockout clones without the detection of 5′ or 3′ sites ligation between the genomic region and the cassette; %KI, percentage of knock-in in 93 clones.

Conventional targeting vectors consist of selection markers flanking homology arms with the target genomic regions^12^. To compare the KO efficiency of the ssODNs and the homology arms, we constructed a DNA fragment coding a neomycin resistance gene (NeoR) which flanked the 250 bp homology arms (Fig. 4). While ssODNs with the cassette coding NeoR provided low KO efficiency, compared with other antibiotics (Fig. 2A), the homology arms ligated to the cassette coding NeoR significantly increased the KI efficiency of the selection marker, resulting in 8 KO clone, and 5 KO clones without the expected ligation at both 5′ and 3′ sites (63.4% for 5′ and 64.5% for 3′, Figs. 4 and S4). We confirmed the homologous recombination in 54 out of 59 clones for 5′ and in 59 out of 60 clones for 3′ by sequencing PCR fragments (Fig. S4). The results indicate that the homology arms increase the KI efficiency under the low antibiotic dose selection.

### Multiple selection markers did not increase the efficiency of obtaining KO clones

**Figure 5:**
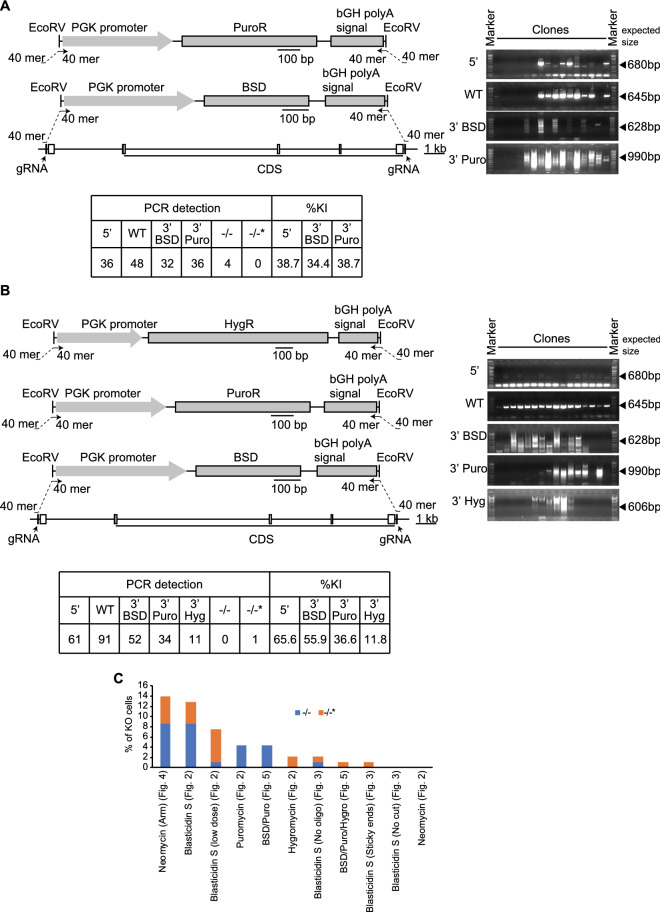
Multiple selection markers did not increase the efficiency to obtain KO clones. (**A**, **B**) The schemas of knockout of the target gene by pX330 plasmids, ssODNs, and the cassette with PuroR and BSD as selection markers (**A**) or with HygR, PuroR, and BSD as selection markers (**B**). The concentration of antibiotics for selection is 100 µg/mL BSD and 5 µg/mL Puromycin (**A**) or 100 µg/mL BSD, 5 µg/mL Puromycin, and 400 µg/mL Hygromycin (**B**). Agarose gel images show the genotyping results. The table indicates the genotyping results of 93 clones. BSD, blasticidin S resistant gene; HygR, hygromycin resistant gene; PuroR, puromycin resistant gene; ssODNs, single-strand oligodeoxynucleotides. 5′, 5′ site between the genomic region and the cassette; 3′, 3′ site between the genomic region and the cassette; WT, wild-type allele; −/−, homozygously knockout clones: −/−*, homozygously knockout clones without the detection of 5′ or 3′ sites ligation between the genomic region and the cassette; %KI, percentage of knock-in in 93 clones. (**C**) Bar graph of KO percentage comparing different approaches in this study.

A previous study suggested using two antibiotic resistance genes as selection markers increases in homozygous KI clones from human HCT116 cells^13^. Based on the study, we hypothesized that 2 cassettes may increase the efficiency in generating KO clones. Antibiotics selection with puromycin and blasticidin S increased the efficiency to knock cassettes with selection markers into the target genomic regions (38.7% for 5′, 34.4% for 3′ blasticidin S resistant gene (BSD), and 38.7% for 3′ puromycin resistant gene (PuroR), Figs. 5A and S5A). However, genotyping showed aberrant fragment sizes and multiple amplicon bands in almost of all clones (Figs. 5A and S5A). Thus, in our study, two antibiotics selection approach did not increase the efficiency of obtaining KO clones, compared with single antibiotic selection. We further attempted to generate KO clones with three antibiotics selection with hygromycin, puromycin, and blasticidin S (Fig. 5B). The three antibiotics selection strategy decreased the efficiency to knock the cassettes into the target genomic regions (65.6% for 5′, 55.9% for 3′ BSD, 36.6% for 3′ PuroR, 11.8% for hygromycin resistant gene (HygR), Figs. 5B and S5B), compared with the 2 antibiotics selection. Moreover, three antibiotics selection approach decreased the efficiency to establish KO clones as well (Fig. 5B). The genotyping results showed aberrant fragment sizes and amplicon bands in almost all clones, similar to two antibiotics selection approach (Figs. 5A,B, S5A,B). Collectively, a bar graph of KO percentages comparing different approaches indicates that multiple selection markers do not promote generation of KO clones using SUCCESS (Fig. 5C).

### SUCCESS generated *Antxr2* knockout clone in ID8 murine ovarian cancer cell line

**Figure 6:**
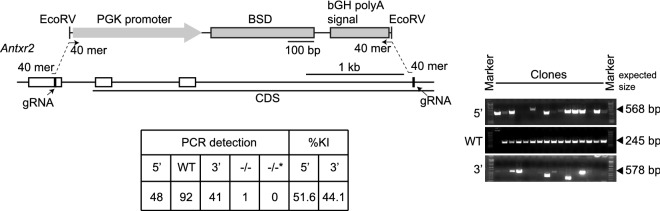
Evaluation of SUCCESS to target *Antxr2* in murine ovarian cancer cell line ID8-luc2. The schema of knockout of *Antxr2* gene by SUCCESS is shown. Agarose gel images show the genotyping results. The table indicates the genotyping results of 93 clones. 5′, 5′ site between the genomic region and the cassette; 3′, 3′ site between the genomic region and the cassette; WT, wild-type allele; −/−, homozygously knockout clones: −/−*, homozygously knockout clones without the detection of 5′ or 3′ sites ligation between the genomic region and the cassette; %KI, percentage of knock-in in 93 clones.

SUCCESS was sufficiently used to delete a target genomic region other than *Apod* in a different cell line. We designed gRNAs and ssODNs to delete a part of *Antxr2*, in the murine ovarian cancer cell line ID8 (Fig. 6). The integration of the cassette into the target genomic region in *Antxr2* was detected following 200 µg/mL blasticidin S selection (51.6% for 5′, 44.1% for 3′), resulting in 1 knockout clone (Figs. 6 and S6). Taken together, the data indicated that SUCCESS is a useful method to edit the genome in multiple cancer cell lines.

## Discussion

KO/KI of a gene of interest is an effective approach to understand its function. Several genome editing methods have been developed to generate KO or KI clones in cell lines and embryos of various species^14–16^. To generate KO clones, replacing the full length of the gene of interest with the selection marker is the most reliable method. However, constructing a targeting vector is time-consuming. Due to polyploidy of cancer cells, a highly efficient method is required to swap the target genomic region with the selection marker to generate KO clones. In the present study, we developed SUCCESS to generate KO clones using two plasmids coding Cas9 and gRNA, two 80mer ssODNs, and a blunt-end universal cassette with selection marker. High concentration of antibiotics increased in the probability to select KO clones. Thus, it is critical to determine antibiotics that do not affect cell morphology at the 2 ~ 25 times concentration of antibiotics, compared to the concentration of antibiotics to kill almost all parental cells. We confirmed the efficiency of our novel method by generating KO clones of two target genes in two cancer cell lines. This simple method allows to generate KO clones in cell lines expeditiously.

Several antibiotics were used to select cells with a selection marker. Neomycin is widely used to generate KO clones in embryonic stem cells. In B16F10 cells, neomycin was not sufficient to select KO clones (Fig. 2), although higher neomycin doses may promote selection of KO clones. In the present study, we did not examine neomycin concentration > 4000 µg/mL, since too high concentrations of antibiotics may change cell characteristics and/or morphology. Instead, selection with blasticidin S 100 µg/mL showed the highest efficiency of selecting KO clones in B16F10 cells. These cells may require many copies of an antibiotic resistance gene to acquire resistance against blasticidin S, compared to other antibiotics. Indeed, low blasticidin S concentration (5 µg/mL) significantly decreased the efficiency to select KO clones, suggesting that the probability of selecting KO clones is increased by using an antibiotic, acquiring resistance against which requires multiple copies of the antibiotic resistance gene. Thus, appropriate antibiotics for selecting KO clones may be cell-dependent. Although KO clones can be selected without a selection marker^17^, the probability of selecting KO clones is dependent on gRNA activity to cut the target genomic regions and on the efficiency of DNA recombination. These offers an advantage of using a selection marker to establish KO clones.

Random integration of the selection marker may disrupt the function of non-target genes. Recently, CRISPR/Cas9 system sometimes causes genome instability^18^, off-targets from the genome manipulation^19^, reading frame restoration^20^, and gene conversion^21^. Although our method could delete a long genomic region from all alleles, which prevents reading frame restoration, loss-of-heterozygosity, and gene conversion, our method also had the possibility of off-target gRNA effects. Thus, rescue experiments may be important to prevent the possibility of a phenotype caused by an undesired KO^17^.

Our results suggest that higher concentrations of antibiotics increase the probability of selecting clones with KIs carrying the selection marker (Fig. 3). Un-cut plasmids carrying a selection marker can generate stably-expressed clones. In our study, high blasticidin S concentration (100 µg/mL) did not allow cells to acquire antibiotic resistance by the un-cut plasmid carrying the selection marker, although the plasmid had the ability to express antibiotic resistant genes with random integration. These results indicate that high antibiotic concentration increases the probability of obtaining KO clones.

Our results also suggested that the shape of the DNA ends of the cassettes with the selection marker influences the efficiency to select KO clones (Figs. 2 and 3). The blunt-ends DNA of the cassette with selection marker showed high efficiency to select KO clones, compared to the sticky ends of the cassette with selection marker. As Cas9 generates the blunt-ends by double-strand breaks^1–3^, the blunt-ends of the cassette with selection marker may be easily ligated to the blunt ends of the genomic DNA.

The 80mer ssODNs acted as homology arms to integrate the cassette with selection marker with the target genomic region cut by Cas9 (Fig. 3). ssODNs have homology sequences, at the end of the cassette as well as at the end of the genomic DNA. ssODNs probably enhanced ligation between the ends by activating microhomology-mediated end joining (MMEJ)^22^, because NHEJ inhibitor did not decrease in the survived cell number after high dose antibiotic selection in our method, meaning that NHEJ may have the limited role in the ligation^23,24^. The 250 bp homology arm-flanked selection marker increased the efficiency of selecting KO clones, compared to 80mer ssODNs (Figs. 2 and 4). Previously, we generated GFP KI clones in the Androgen receptor gene using an ~ 250 bp homology arm-flanked targeting vector^25^. Although the targeting vectors increased the efficiency to select KO clones, 80mer ssODNs provided comparable selection efficiency to select KO clones with appropriate antibiotic selection.

Selection using multiple selection markers has been reported to increase the efficiency of obtaining KO/KI clones^13^. However, in our study with B16F10 cells, selection using multiple antibiotics did not improve the probability for selecting KO clones (Fig. 5). The integration pattern of the cassette into the genome was disordered with multiple antibiotics selection, compared to single antibiotic selection. The data suggests that selection with multiple antibiotics prevents the DNA ligation between the cassette with selection marker and genomic DNA. Since cells were re-seeded after 5 days of antibiotics selection to create single cell colonies, almost all DNA ligation between the cassette and genome was expected to be completed in this period. Indeed, single antibiotic selection did not cause disordered DNA ligation between the cassette and genome (Figs. 2 and S2). Our results are consistent with slow rates in repairing double-strand breaks induced by Cas9^26^. Although there is a possibility that the multiple antibiotics selection works well in different cell lines, our data indicate that multiple antibiotics selection is not appropriate when using SUCCESS to generate KO/KI clones.

## Conclusion

In summary, SUCCESS is a reliable approach to easily obtain KO clones by introducing two plasmids coding Cas9 and gRNA, two 80mer ssODNs, and a blunt-end universal cassette with selection marker under high antibiotic selection. Since SUCCESS does not require construction of a targeting vector, this facilitates easy generation of KO/KI cell lines.

## Methods

### Cell culture

B16F10 murine melanoma cells were purchased from the American Type Culture Collection. B16F10 cells were cultured in Dulbecco’s Modified Eagle’s Medium (DMEM) (Nacalai Tesque) supplemented with heat inactivated 10% fetal bovine serum (FBS) (Biowest), 100 U/mL penicillin, and 100 µg/mL streptomycin (Penicillin–Streptomycin Mixed Solution) (Nacalai Tesque). ID8-luc2 murine ovarian cancer cells were a gift from the Department of Obstetrics and Gynecology, Osaka University Graduate School of Medicine. ID8 luc2 cells were cultured in Roswell Park Memorial Institute (RPMI) (Nacalai Tesque) supplemented with heat inactivated 10% FBS, 100 U/mL penicillin and 100 µg/mL streptomycin, at 37 °C and 5% CO_2_ in the CO_2_ incubator. NHEJ was inhibited by 1 µM NHEJ inhibitor-SCR7 following manufacture’s instructions (Xcess Biosciences Inc.) after electroporation using the NEON system (Thermo Fisher Scientific).

### gRNA design

Two guide RNAs (gRNAs) were designed using DESKGEN (https://www.deskgen.com/landing/). One gRNA specifically recognized the upstream of 5′ UTR of *Apod*. This gRNA was selected by the given score of 75 and the specificity in DESKGEN system. Another gRNA with a score of 67 specifically recognized the downstream from stop codon of *Apod*. 5′ gRNA sequence is 5′-ACTTACAAGAGCATTCTGAG-3′. The 3′ gRNA sequence is 5′-TCTTAAGTACCAAGCAACCA. These gRNA sequences were cloned into the pX330 vector (addgene: #42230).

### Generation of KO cells

The NEON system was used to electroporate 2.5 µg DNA, consisting of 500 ng each of two pX330 coding Cas9 and gRNA, ~ 500 ng each of 80mer ssODNs, and 500 ng of the blunt-end DNA coding selection marker cut by EcoRV into 3 × 10E5 B16F10 cells with the following setting: voltage, 1100; width, 30 pulse; number, 1, or ID8-luc2 with the following setting: voltage, 1400; width, 30 pulse; number, 1, and following the manufacturer’s instruction. AscI and NotI were used to generate the sticky ends of DNA coding selection marker. For multiple antibiotics selection, the same amount of DNA was used for electroporation. To determine a suitable concentration of antibiotics, we first examined the concentration of antibiotics to kill almost all parental cells. We next added 2 ~ 25 times concentration of antibiotics, compared to the dose of antibiotics for the parental cells, at four different points for selecting KO clones after electroporation of pX330 plasmids, 80mer ssODNs, and the blunt-ended selection marker fragment. The transfected cells were cultured under the indicated antibiotic selection for 5 days from the next day of electroporation. The selected 3000 cells at the highest dose of antibiotics without changing morphology were re-seeded on 10 cm dish and were cultured for 8 days in the presence of following antibiotic concentration: 20 µg/mL blasticidin S, 1 µg/mL puromycin, 2 mg/mL neomycin, and 100 µg/mL hygromycin. Simultaneously, bulk genome was extracted from a part of the cell suspension washed with phosphate buffered saline (PBS) and lysed in 50 mM NaOH, heated at 95 °C for 10 min, then neutralized by 1/10 volume of 1 M Tris–HCl pH 8.0. Single colonies were transferred to the wells of 96-well plate. One third of the sub-confluent cells were used for the DNA extraction, while the remaining cells were frozen by using CELL BANKER (NIPPON ZENYAKU KOGYO). Genotyping PCR was performed using KOD FX Neo (TOYOBO). The primer sequences are listed in Supplementary Table S1. The PCR amplicons were analyzed by electrophoresis on a 2% (w/v) agarose gel and visualized by staining with ethidium bromide. For direct sequencing, the PCR amplicons were purified by Monarch DNA Gel Extraction Kit (New England Biolabs) and analyzed using Big Dye (Thermo Fisher Scientific).

## Supplementary Information

Supplementary Information 1.

Supplementary Table 1.

## Data Availability

All data generated or analyzed during this study are included in this published article and its supplementary information files.
