# Supplementary material for: A simple method using CRISPR-Cas9 to knock-out genes in murine cancerous cell lines
Source: Sci Rep. 2020 Dec 18;10:22345. doi: 10.1038/s41598-020-79303-0 (PMC7749119; doi:10.1038/s41598-020-79303-0)

SUPPLEMENTAL DATA

A simple method using CRISPR-Cas9 to knock-out genes in murine cancerous cell lines

Airi Ishibashi, Kotaro Saga, Yuuta Hisatomi, Yue Li, Yasufumi Kaneda, and Keisuke Nimura

INDEX OF SUPPLEMENTAL MATERIALS

Figure S1, relates to Figure 2: *Antibiotic selection concentration to remove wild type B16F10 cells.*

Figure S2, relates to Figure 2: *Genotyping results and the effect of NHEJ inhibitor.*

Figure S3, relates to Figure 3: *Genotyping results*

Figure S4, relates to Figure 4: *Genotyping results*

Figure S5, relates to Figure 5: *Genotyping results*

Figure S6, relates to Figure 6: *Genotyping results*

Table S1: *The primers used in the study.*

SUPPLEMENTARY FIGURE LEGENDS

Figure S1. Antibiotic selection concentration to remove wild type B16F10 cells related to Figure 2. Wild type B16F10 cells were treated in the medium containing the indicated concentration of antibiotic.

Figure S2. Genotyping results related to Figure 2 and the effect of NHEJ inhibitor. (**A**-**D**) Agarose gel images show the results of genotyping. WT, wild type; Bulk, the

extracted genome from bulk cells; DW, distilled water. (**E**) The bar graph shows survived cell number after 100 μg/ml blastcitidin S selection with 1μM NHEJ inhibitor-SCR7 or DMSO control for 5 days. Live cells were counted with trypan-blue. n = 4

Figure S3. Genotyping results related to Figure 3. (**A-B**) Agarose gel images show the results of genotyping. WT, wild type; Bulk, the extracted genome from bulk cells; DW, distilled water. Rectangles show the cropped area for figure 3A and 3B.

Figure S4. Genotyping results related to Figure 4. Agarose gel images show the results of genotyping. WT, wild type; Bulk, the extracted genome from bulk cells; DW, distilled water. Rectangles show the cropped area for figure 4.

Figure S5. Genotyping results related to Figure 5. (**A-B**) Agarose gel images show the results of genotyping. WT, wild type; Bulk, the extracted genome from bulk cells; DW, distilled water. Rectangles show the cropped area for figure 5A and 5B.

Figure S6. Genotyping results related to Figure 6. Agarose gel images show the results of genotyping. WT, wild type; Bulk, the extracted genome from bulk cells; DW, distilled water. Rectangles show the cropped area for figure 6.

Table S1. The primers used in the study.

Figure S1

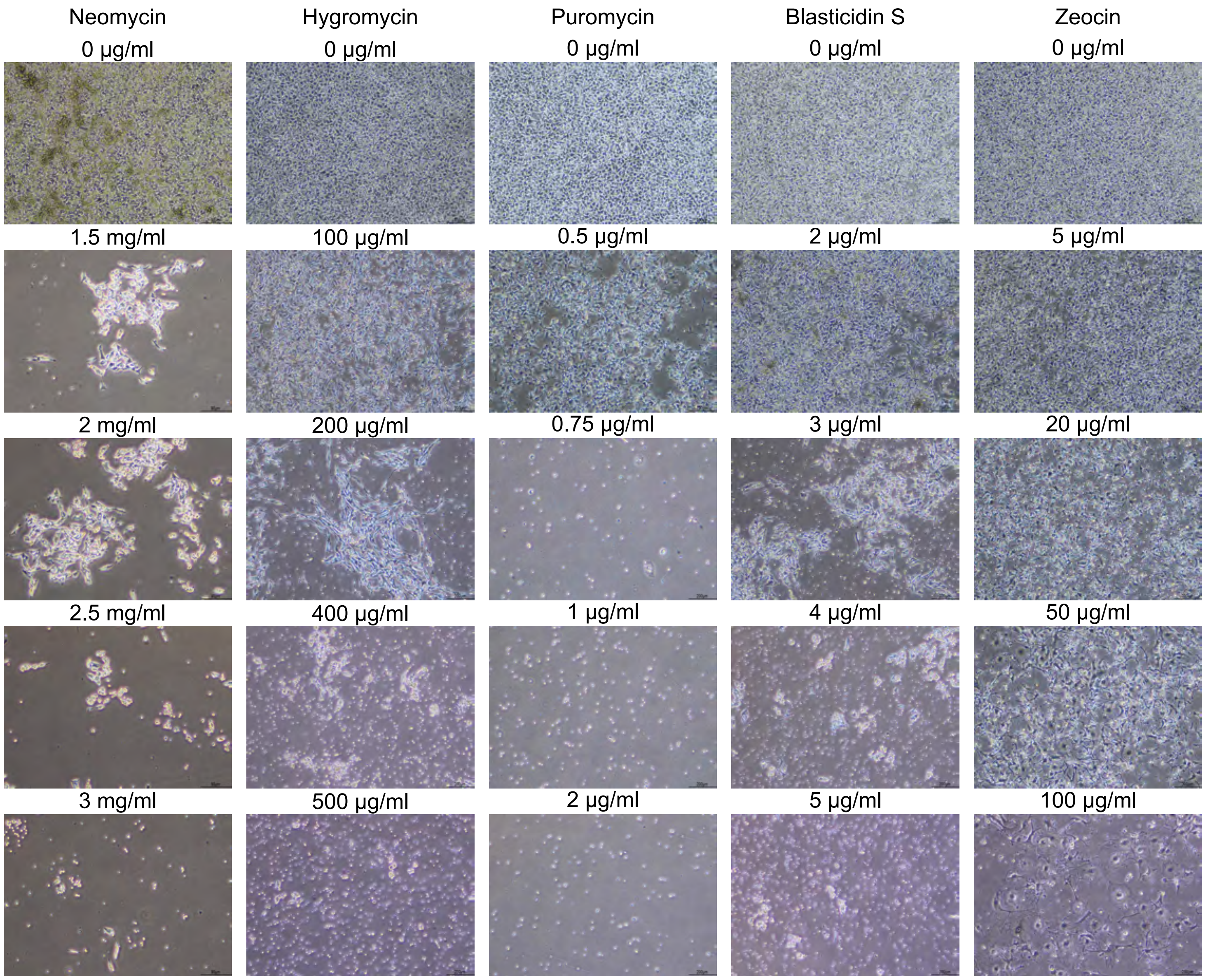
Neomycin
Hygromycin
Puromycin
Blasticidin S
Zeocin
0 µg/ml
0 µg/ml
0 µg/ml
0 µg/ml
0 µg/ml
1.5 mg/ml
100 µg/ml
0.5 µg/ml
2 µg/ml
5 µg/ml
2 mg/ml
200 µg/ml
0.75 µg/ml
3 µg/ml
20 µg/ml
2.5 mg/ml
400 µg/ml
1 µg/ml
4 µg/ml
50 µg/ml
3 mg/ml
500 µg/ml
2 µg/ml
5 µg/ml
100 µg/ml

Figure S2

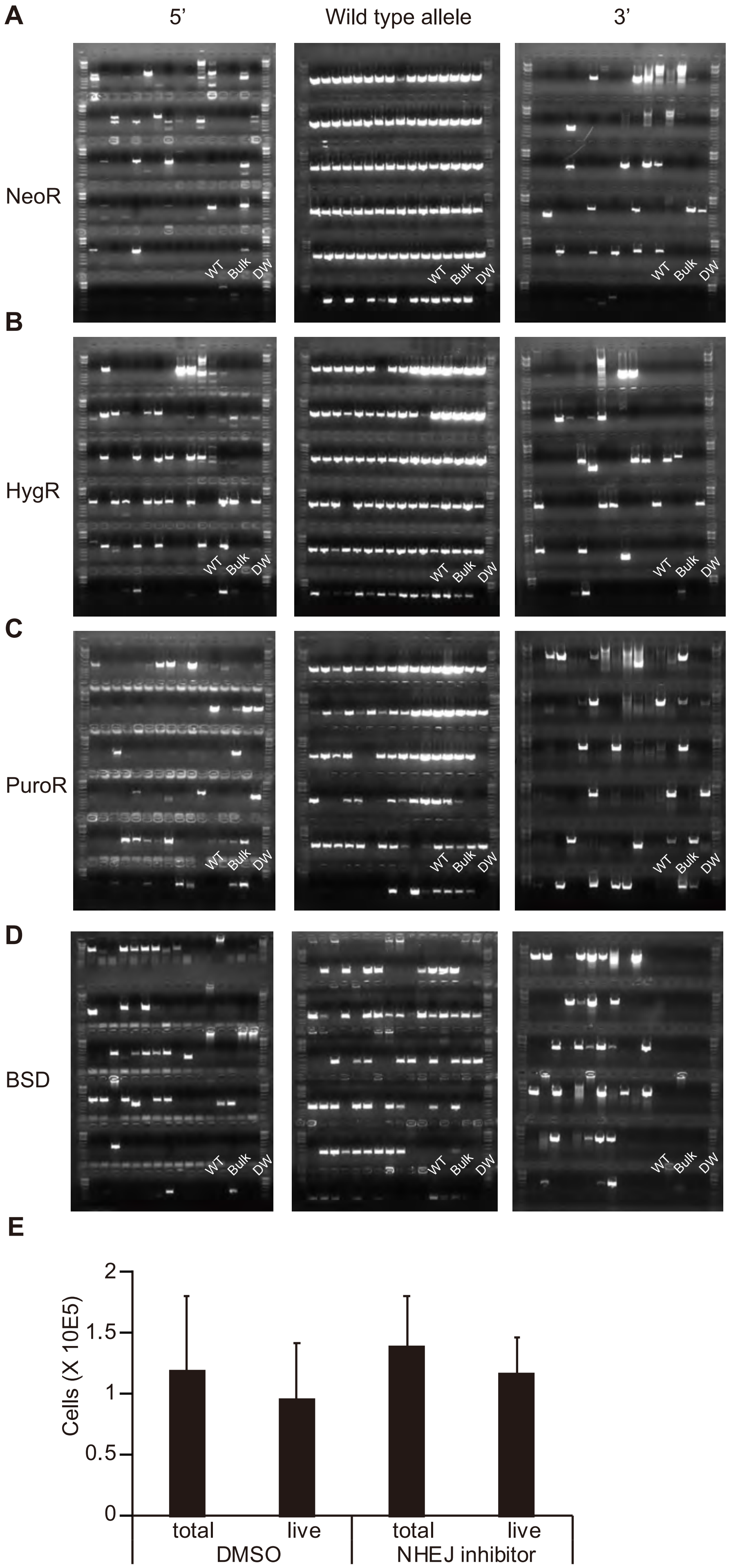
A
5'
Wild type allele
3'
NeoR
WT
Bulk
DW
B
HygR
C
PuroR
D
BSD
E
Cells (X 10E5)
2
1.5
1
0.5
0
total
live
total
live
DMSO
NHEJ inhibitor

Figure S3

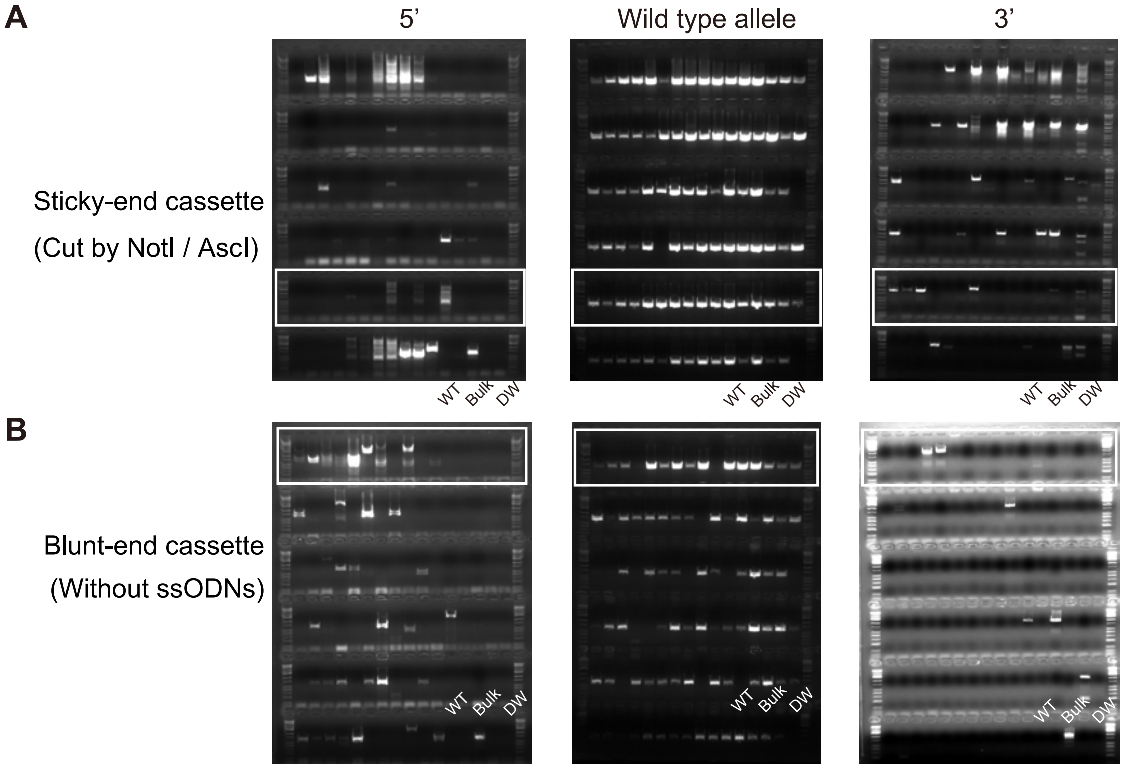
A
5’
Wild type allele
3’
Sticky-end cassette
(Cut by NotI / AscI)
WT
Bulk
DW
B
Blunt-end cassette
(Without ssODNs)

Figure S4

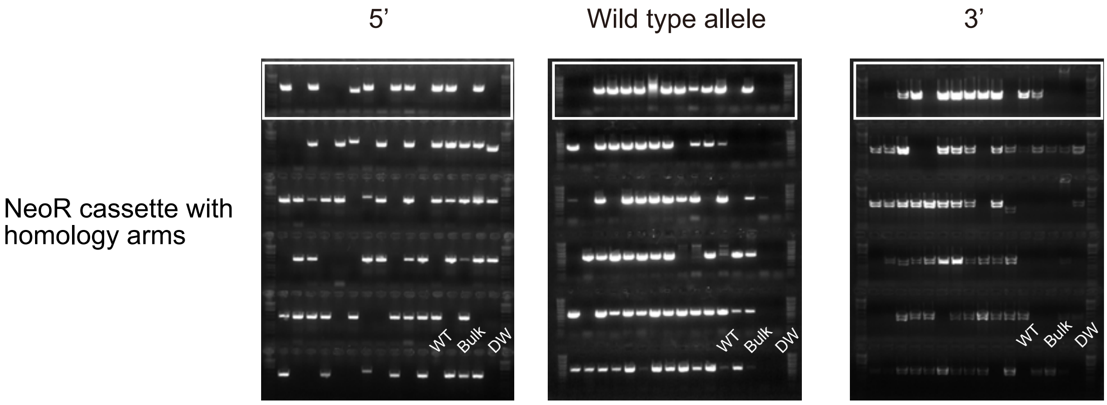
5’
Wild type allele
3’
NeoR cassette with homology arms
WT
Bulk
DW
WT
Bulk
DW
WT
Bulk
DW

Figure S5

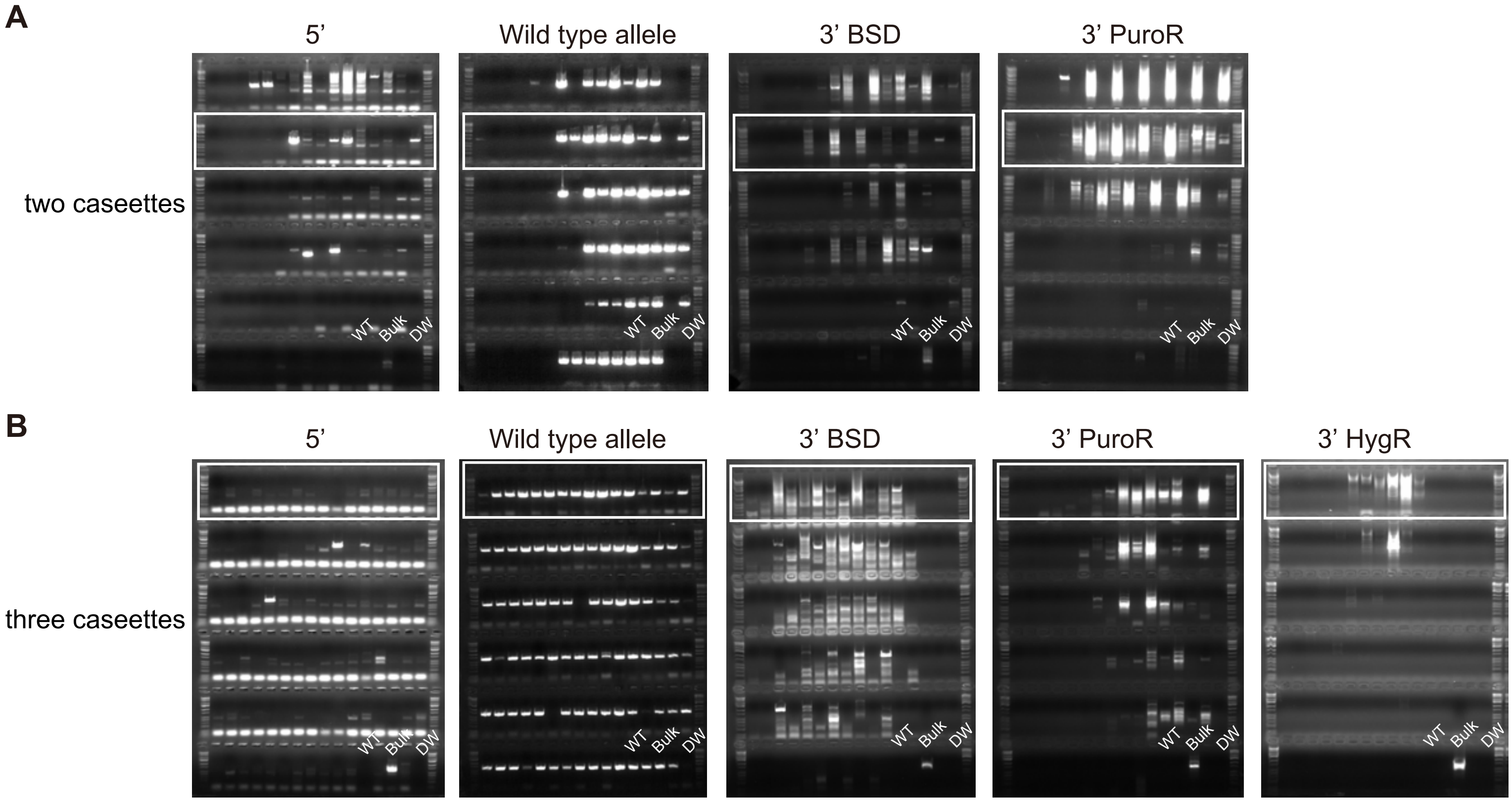
A
5’
Wild type allele
3’ BSD
3’ PuroR
two caseettes
WT
Bulk
DW
B
5’
Wild type allele
3’ BSD
3’ PuroR
3’ HygR
three caseettes
WT
Bulk
DW

Figure S6

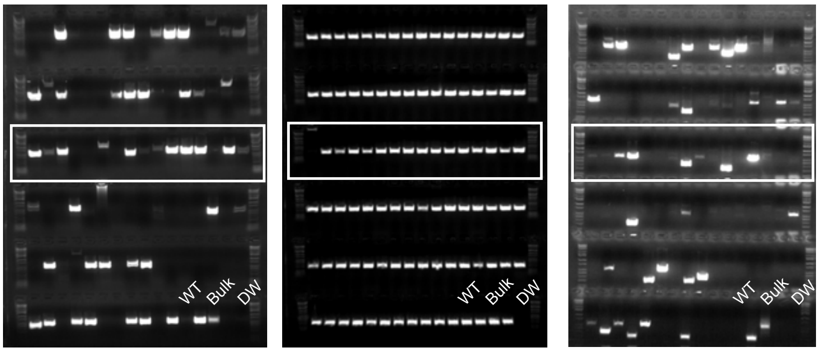

Supplement: Supplementary file 1 — Supplementary Information 1. [file 41598_2020_79303_MOESM1_ESM.pdf]
