# Supplementary material for: A simple method using CRISPR-Cas9 to knock-out genes in murine cancerous cell lines
Source: Sci Rep. 2020 Dec 18;10:22345. doi: 10.1038/s41598-020-79303-0 (PMC7749119; doi:10.1038/s41598-020-79303-0)
Supplement: Supplementary file 2 — Supplementary Table 1. [file 41598_2020_79303_MOESM2_ESM.pdf]

Supplementary table 1

B16F10	forward(5'→3')	reverse(5'→3')
5'	CCACAACGTCTGTCTTCACAAT	TACTTCCATTTGTCACGTCCTG
5'(Arm for Fig4)	GGTCTGTGGTATGCCCTAATGT	TACTTCCATTTGTCACGTCCTG
WT	CTGGACCTTATCCAAACTGGAG	GTTTGGCTTCACCCTTTACTTG
3'(BSD)	GCCATAGTGAAGGACAGTGATG	CTAACTTGGCCATCAGCTTTCT
3'(Puro)	AGCTGCAAGAACTCTTCCTCAC	CCCAGATATTTTCAGGATGCTC
3'(Hygro)	AGTACTCGCCGATAGTGGAAAC	CTAACTTGGCCATCAGCTTTCT
3'(Neo)	CTTCCTCGTGCTTTACGGTATC	CCCAGATATTTTCAGGATGCTC

ID8	forward(5'→3')	reverse(5'→3')
5'	CGGGAGAGTTGGATATGTTTAG	TACTTCCATTTGTCACGTCCTG
WT	CGAGACTCCAGGGCTACTTG	ATGCAAGCCACATTGGGTAT
3'(BSD)	GCCATAGTGAAGGACAGTGATG	GTGCAGTCTTTTGTTGCAAGTC
